# Functional deficiency of NBN, the Nijmegen breakage syndrome protein, in a p.R215W mutant breast cancer cell line

**DOI:** 10.1186/1471-2407-14-434

**Published:** 2014-06-13

**Authors:** Bianca Schröder-Heurich, Natalia Bogdanova, Britta Wieland, Xiaoxi Xie, Monika Noskowicz, Tjoung-Won Park-Simon, Peter Hillemanns, Hans Christiansen, Thilo Dörk

**Affiliations:** 1 Clinics of Obstetrics and Gynaecology, Hannover Medical School, Carl-Neuberg Straße 1, D-30625 Hannover, Germany; 2 Clinics of Radiation Oncology, Hannover Medical School, Carl-Neuberg Straße 1, D-30625 Hannover, Germany

**Keywords:** Breast carcinoma, DNA damage repair, DNA double strand break repair disorder, Ionising radiation sensitivity, MRN complex

## Abstract

**Background:**

Mutations in NBN, the gene for Nijmegen Breakage Syndrome (NBS), are thought to predispose women to developing breast cancer, but a breast cancer cell line containing mutations in NBN has not yet been described. The p.R215W missense mutation occurs at sub-polymorphic frequencies in several populations. We aimed to investigate its functional impact in breast cancer cells from a carrier of this *NBN* mutation.

**Methods:**

Breast cancer cell lines were screened by immunoblotting for NBN protein levels, and the *NBN* coding region was sequenced for mutation analysis. Radiosensitivity assays and functional studies were performed through immunocytochemistry and immunoblotting, and flow cytometry was employed to assess cell cycle progression. Impedance measurements were used to study the consequences of PARP1 inhibition. Statistical comparisons between cell lines were performed using t-tests.

**Results:**

HCC1395 breast cancer cells exhibited reduced NBN protein levels. Direct sequencing identified the *NBN* p.R215W mutation in the hemizygous state, in addition to a truncation in BRCA1. Mutations in both genes were already present in the heterozygous state in the patient’s germline. HCC1395 cells were highly radiosensitive, susceptible to apoptosis and were deficient in the formation of NBN foci. There was also evidence for some impairment in the formation of γH2AX, MDC1, and 53BP1 foci after irradiation; these foci appeared smaller and irregular compared with repair foci in wild-type cells, although ATM signalling was largely unaffected. In line with their deficiency in NBN and BRCA1, HCC1395 cells were particularly sensitive to PARP1 inhibition.

**Conclusion:**

Our results indicate that the p.R215W mutation in the HCC1395 breast cancer cell line impairs NBN function, making this cell line a potentially useful cellular model for studying defective NBN protein within a mutant BRCA1 background.

## Background

Breast cancer is a genetically heterogeneous disease, and several predisposing genes are involved in DNA double strand break repair [1-3]. One of these is *NBN*, the gene for Nijmegen Breakage Syndrome (NBS) [4-6]. NBS is an autosomal recessive chromosomal instability disorder characterised by microcephaly, stunted growth, immunodeficiency, a high cancer predisposition and a marked sensitivity towards ionising radiation [7]. The syndrome is most common in Eastern Europe due to a Slavic founder mutation, c.657del5, in the *NBN* gene [4]. This gene encodes a 754 amino-acid protein named NBN, p95 or nibrin, that interacts with the MRE11 and RAD50 proteins in sensing DNA damage and aids to recruit the ataxia-telangiectasia mutated kinase, ATM, to the sites of DNA double-strand breaks [5,8]. It further interacts at the DNA damage sites with phosphorylated histone H2AX (γH2AX) through its tandem breast cancer carboxy-terminal (BRCT) domain [9,10]. Loss of NBN function leads to radioresistant DNA synthesis and deficiencies in proper DNA double-strand break repair [11,12].

While biallelic mutations in the *NBN* gene give rise to NBS, monoallelic mutations have been found to predispose the heterozygous carriers within NBS families towards malignancies [13]. Furthermore, an increased frequency of the most common NBN mutation c.657del5 has been observed in Eastern European breast cancer patients compared with healthy controls [14-19]. A missense mutation, p.R215W, in the tandem BRCT domain has been suggested as a more wide-spread candidate breast cancer susceptibility allele [15,19]. Compound heterozygosity of the p.R215W substitution with the c.657del5 mutation has been reported in NBS patients with severe disease [20]. *In vitro* mutagenesis studies indicated that p.R215W might be a functional mutation that impairs the association of NBN with γH2AX [21]. However, further cellular models for this missense mutation would be useful to fully clarify its role in breast cancer.

In the present study we report on the identification of a breast cancer cell line, HCC1395, that harbors p.R215W in the hemizygous state, and we investigate the functional competence of the mutant NBN protein in this cell line.

## Methods

### Cell culture

Cell lines were obtained from the American Type Culture Collection (ATCC) in 2010. Human breast cancer epithelial cell lines HCC1395 and HCC1937 were cultured in RPMI 1640 with 10% fetal calf serum, 500 U/ml penicillin, 0.5 mg/ml streptomycin and 2 mM L-Glutamine. Lymphoblastoid cells HCC1395 BL were cultured in RPMI1640 with 15% fetal calf serum and supplements as above. Human normal breast epithelial MCF10A cells were cultured in MEBM, supplemented with MEGM Single Quots according to the manufactures instruction (Lonza). All cells were grown at 37°C in a humidified atmosphere supplemented with 5% CO_2._ Ionizing radiation (IR) with doses between 0.1 – 6 Gy was applied to the cells using a Mevatron MD-2 accelerator (Siemens, Munich, Germany). Olaparib was purchased from LC Laboratories (Woburn, MA, USA), dissolved in DMSO and stored at -20°C before usage.

### Genetic analysis

Genomic DNA was extracted from the cultured breast cancer epithelial cells using proteinase K digestion and phenol-chloroform extraction. The coding region of *NBN* and selected regions of *BRCA1* (exon 20), *BRCA2* (exon 11) and *TP53* (exon 5) gene were amplified by PCR using the primer pairs given in Additional file 1: Table S1, and purified PCR products were subjected to direct sequencing using BigDye v1.1 terminator chemistry and a 3100 Avant capillary sequencer (Life Technologies). Sequencing data were analyzed with the Sequencing Analysis 5.1.1 software.

### Colony formation assay

Cells were seeded in six-well plates and, after 24 hours incubation, were irradiated with doses between 0–6 Gy. Radiation doses between 0.1-1 Gy or between 2–6 Gy were applied in different experiments. Plating efficiency was consistently lower for HCC1395 cells than for MCF10A cells (about 58% for MCF10A compared with about 2% in HCC1395). Medium was gently changed every two days. After 5–7 days incubation for MCF10A and 10–12 days incubation for HCC1395, colonies were fixed with 3% (w/v) PFA, 2% (w/v) sucrose in PBS for 10 min, stained with 0.5% (w/v) crystal violet and counted by microscopy. Surviving colonies were counted as positive above a threshold of 50 cells. The survival fraction (SF) of irradiated cells was expressed as a percentage of colonies per seeded cells after normalisation by the plating efficiency of unirradiated cells. Each experiment was performed at least 3 times with both cell lines.

### Lysate preparation and immunoblotting

For preparation of protein extracts, cells were lysed in cell extraction buffer (50 mM Tris pH 7,4, 150 mM NaCl, 2 mM EGTA, 2 mM EDTA, 25 mM NaF, 0.1 mM Na_3_VO_4_, 0.1 mM PMSF, 2 mg/ml Leupeptin, 2 mg/ml Aprotinin, 0.2% Triton X-100, 0.3% NonidetP-40) for 30 min on ice. Protein extracts were cleared through centrifugation at 16100 rcf for 15 min, and supernatants were separated through SDS-PAGE and transferred to nitrocellulose membranes. Antibodies to NBN, SMC1-pS966, KAP1-pS824 were obtained from Novus Biologicals (rabbit polyclonal); anti CHEK2-pS19 from Cell Signaling (rabbit polyclonal); anti RAD50 (mouse monoclonal) from Abcam; anti MRE11 (mouse monoclonal 12D7) from GeneTex; PARP1 and cleaved PARP1 (rabbit polyclonal) from Cell Signaling, and anti β-Actin (mouse monoclonal) from Sigma. Anti-mouse and anti-rabbit horseradish peroxidases labelled secondary antibodies were purchased from GE Healthcare. Visualization of immunoreactive bands was performed by using ECL (Thermo Scientific/Pierce), and their intensity was determined using ImageJ quantitation software.

### Immunocytochemistry

Cells grown on cover glasses in six-well plates were fixed with 3% (w/v) PFA, 2% (w/v) Sucrose in PBS for 10 min. Cells were permeabilized with 0.2% (v/v) Triton X-100 in PBS. Antibodies against NBS1 (Novus Biologicals), MDC1 (Abcam), Histone H2A.x Phospho (S139) (Millipore) and 53BP1 (Bethyl Laboratories) were incubated in 2% (w/v) normal goat serum (Dianova) for 2 hrs. After PBS washing, cells were incubated with FITC-conjugated anti-mouse IgG antibody (Dianova), Alexa Fluor anti-mouse IgG 488 or Alexa Fluor anti-rabbit IgG 546 (Invitrogen) for 1.5 hrs. DNA was counterstained with DAPI (Invitrogen) and cells were mounted with ProLong® Gold (Invitrogen). Foci were counted under a Leica DMI6000B microscope, and results from four independent experiments were statistically analysed using GraphPad Prism 4 with a t-test. P values below α < 0.05 were considered significant. For a more detailed inspection of the size and area of foci, images were taken as z-stacks by using a Leica TCS SP2 confocal microscope (40× or 63× magnification) and image acquisition was carried out using CorelPhotoPaintX4 Software and evaluated using ImageJ software. The number of pixels was determined as a proxy for the foci area.

### Flow cytometry

For DNA content analysis cells were irradiated with 6 Gy, were fixed in 70% ethanol after the indicated time-points (24, 48, 72, 96 hours) and were stained with propidium iodide over night at 4°C. Data acquisition and analysis were performed with a FACS Calibur (BD) flow cytometer and Summit V5.1 software (Beckman Coulter). Results from three independent experiments were analysed using GraphPad Prism 4.

### Impedance measurements

For impedance measurements we used the xCELLigence System (Roche) that provides a highly sensitive method to measure the cell index as a proxy of cell adhesion, proliferation, cell death and morphological alterations. Cell lines were subconfluent, and each cell line has been optimized in previous titration experiments so that cell type-specific optimal conditions for the seeding rate and confluence were obtained for the real-time measurement in these experiments. Cells were seeded at 5000 cells per well in quadruplicates in E-Plate VIEW 96 (Roche) and left untreated for 24 hrs within the RTCA SP station positioned in a 37°C incubator with 5% CO_2_ supply. For monitoring compound mediated cytotoxicity, cells were then treated with DMSO only or with 0.2 μM, 1 μM or 5 μM olaparib and incubated for an additional 72 hrs. The Cell Index was plotted and analysed using RTCA software 1.2.1 (Roche).

## Results

**Figure 1:**
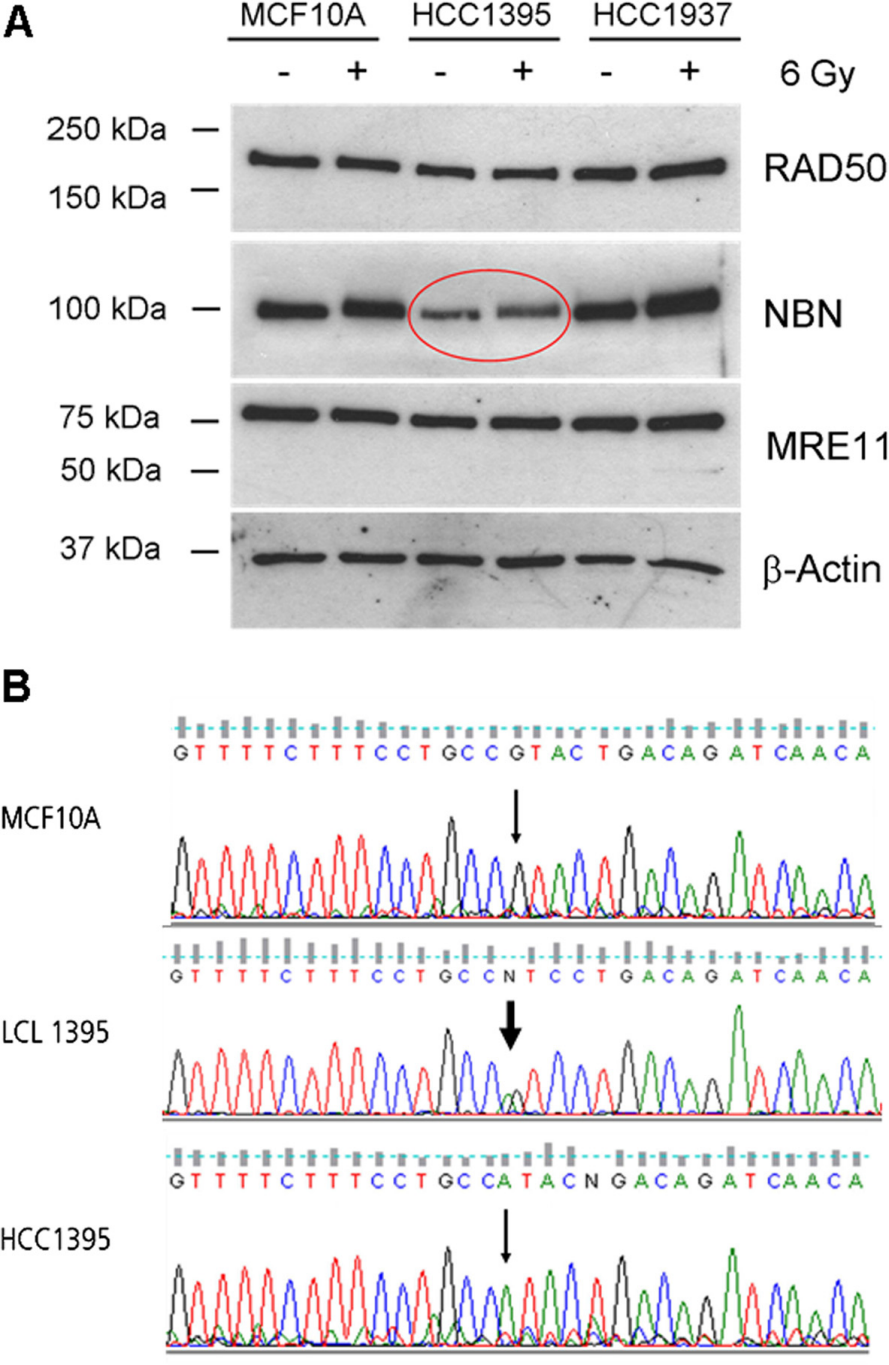
**Identification of a NBN-deficient breast cancer cell line. A**. Immunoblot analysis of MRN complex proteins in a wildtype mammary epithelial cell line (MCF10A) and the breast cancer cell lines HCC1395 and HCC1937, each before and after irradiation (6 Gy, 30 min). HCC1395 cells showed some 70% reduction of NBN while MRE11 and RAD50 immunoreactivity remained largely unchanged. Note the mobility shift in NBN after irradiation that can still be observed in HCC1395 cells at the reduced protein level. **B**. Identification by direct sequencing of the p.R215W (c.643C > T) mutation in genomic DNA from HCC1395 (breast cancer cell line, bottom) and HCC1395 BL (lymphoblastoid cell line, middle) in comparison to wildtype (MCF10A, top). The reverse strand is shown, with the position of the mutation indicated by an arrow. The p.R215W mutation was found in a heterozygous state in HCC1395 BL, and in the apparently homozygous state in HCC1395 (loss of heterozygosity).

We screened by immunoblotting six breast cancer cell lines that are frequently used in molecular cancer research (HCC38, HCC1395, HCC1599, HCC1806, HCC1937, MDA-MB231), for possible deficiencies in the proteins of the MRE11-RAD50-NBN (MRN) complex. One cell line, HCC1395, exhibited significantly reduced levels in NBN that were not seen in the wildtype breast epithelial cell line MCF10A or in the other tested breast cancer lines (Figure 1A). NBN was observed only at 30-40% of wildtype levels in HCC1395 cells, but the protein appeared to be phosphorylated correctly following damage as noted by an electrophoretic mobility shift after irradiation with 6 Gy (Figure 1A).

To identify the molecular basis for the reduced NBN level, the whole NBN coding region was sequenced and the missense mutation p.R215W was uncovered in the hemizygous state (Figure 1B). This result was unexpected since previous genome-wide sequencing studies had not found a *NBN* mutation in HCC1395 cells [22-24]. We therefore investigated lymphoblastoid cells from the same breast cancer patient of whom the HCC1395 cell line had been established. Direct sequencing revealed the p.R215W mutation in the heterozygous state in the lymphoblastoid cells (Figure 1B) indicating that it is a germline mutation and has undergone loss of heterozygosity in the breast tumour cells. The same was confirmed for a mutation in *BRCA1*, p.R1751X, that also was heterozygous in the lymphoblastoid cells and hemizygous in the HCC1395 cell line [24] (Additional file 2: Figure S1). BRCA1 interacts with the MRN complex, however, BRCA1 deficiency could not account for the reduced levels of NBN, since the BRCA1 mutant HCC1937 cell line has normal levels of NBN (Figure 1A).

**Figure 2:**
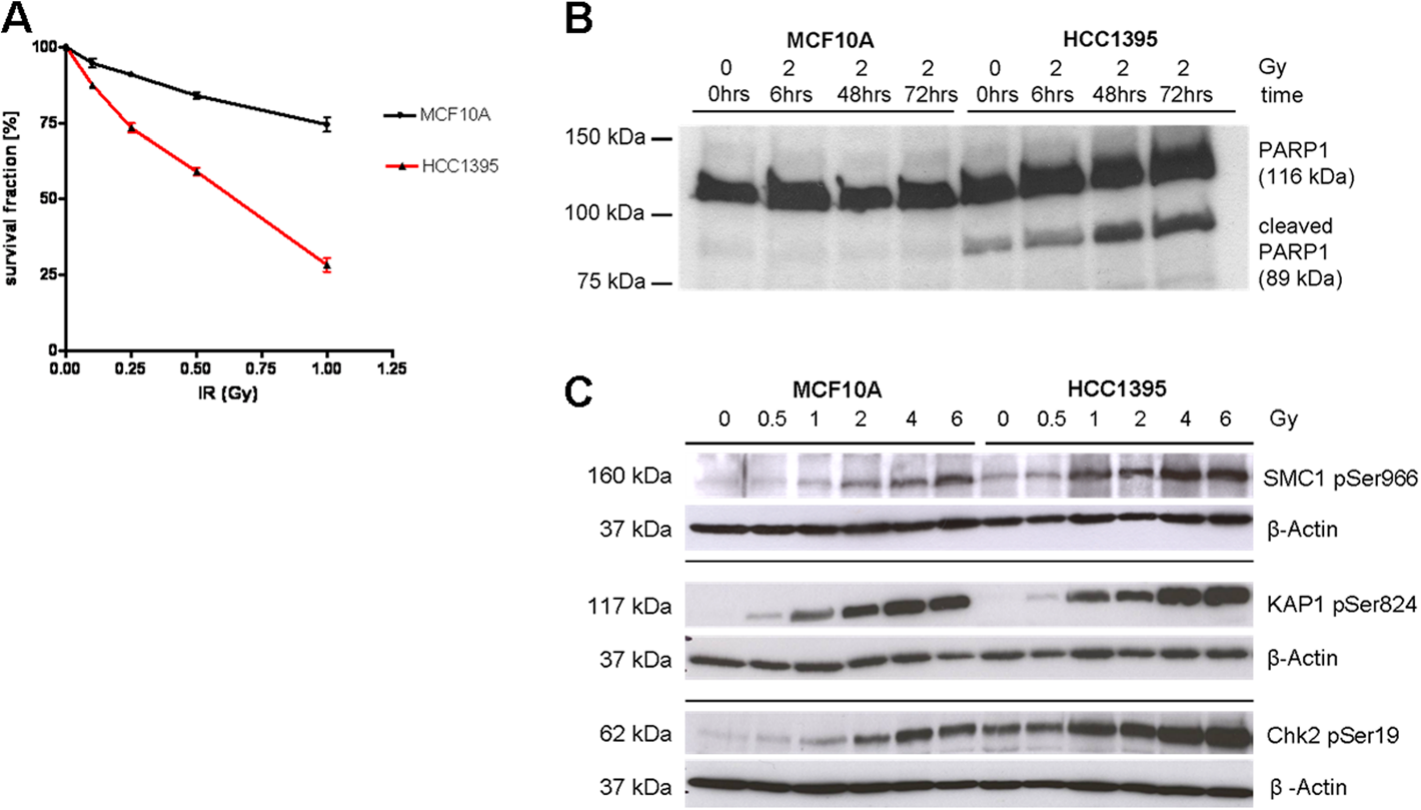
**Assessment of cellular radiosensitivity in the colony formation assay. A**. Cellular radiosensitivity of p.R215W mutant cells (HCC1395) compared with wild type breast epithelial cells (MCF10A) as measured by the colony formation assay after irradiation at doses of 0, 0.1, 0.25, 0.5 or 1Gy. The surviving fraction is presented as the mean value with SEM from at least 3 independent experiments. **B**. Induction of PARP1 cleavage after irradiation with 2 Gy in p.R215W mutant cells (HCC1395) compared with wild type breast epithelial cells (MCF10A) as measured by immunoblotting of cleaved PARP1 (89 kDa) and total PARP1 (116 kDa). **C.** Immunoblot analysis of radiation-induced ATM signalling in HCC1395 cells compared with MCF10A. Cells were untreated or irradiated with 0.5, 1, 2, 4 or 6 Gy as indicated. Protein extracts were prepared 30 min after irradiation and were analysed through Western blotting for their immunoreactivity towards the phosphorylated forms of SMC1 (pSer966, top panel), KAP1 (p824, middle panel), and CHEK2 (pSer19, bottom panel). β-actin served as the loading control in each experiment.

Because NBN deficient cells from NBS patients are highly radiosensitive, we tested whether the p.R215W missense mutation was also associated with an increased sensitivity towards ionising radiation in HCC1395 cells. As shown in Figure 2A and Additional file 3: Figure S2, the HCC1395 cell line was highly radiosensitive in a colony formation assay and could be distinguished from the wildtype response in MCF10A cells even at moderate doses of radiation down to 100 mGy (Figure 2A). This cellular radiosensitivity could not be associated with overt cell cycle abnormalities, such as early G2 accumulation, as judged by flow cytometric analysis of HCC1395 cells 24–72 hrs after irradiation (Additional file 4: Figure S3). However, increased susceptibility to apoptosis was indicated by a markedly increased level of PARP1 cleavage in immunoblots from irradiated HCC1395 cells. PARP1 expression was elevated in HCC1395 cells, and the ratio of cleaved PARP1 versus total PARP1 was about 10% in unirradiated and 30% in irradiated HCC1395 cells whereas cleaved PARP was poorly detectable in MCF10A cells (Figure 2B).

**Figure 3:**
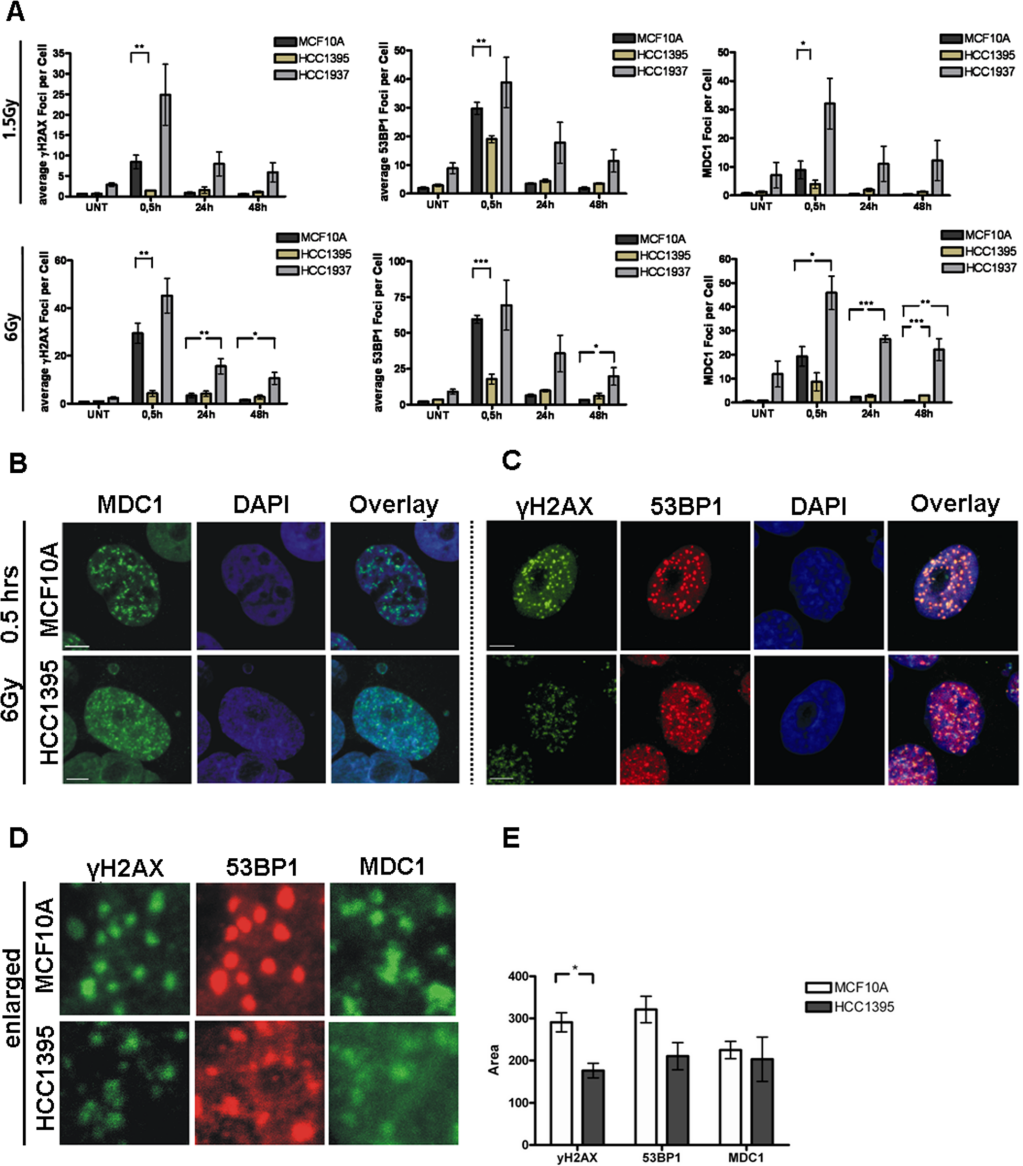
**Immunocytochemical analysis of irradiation-induced repair foci. A**. Evaluation of immunocytochemical evaluation of γH2AX, 53P1 and MDC1 foci after irradiation with either 1.5 Gy (upper panel) or 6 Gy (bottom panel) using conventional fluorescence microscopy. HCC1395 cells were compared with MCF10A cells as a wildtype control and with HCC1937 as a BRCA1 mutant control. Under these assay conditions, the numbers of γH2AX and MDC1 foci appeared significantly reduced in p.R215W mutant cells (HCC1395) at 30 min after irradiation, with 53BP1 foci being slightly reduced. No such reduction was observed in HCC1937 cells. HCC1937 exhibited increased residual levels of foci at 24 hours and 48 hours after irradiation. Data represented as mean & SEM, *p < 0.05, **p < 0.01, ***p < 0.001, n = 4. **B**, **C**. Radiation-induced MDC1 foci **(B)**, γH2AX and 53BP1 foci **(C)**. Representative images are shown for the immunocytochemical analysis of MDC1 (green, Figure B), γH2AX (green, Figure C) and 53BP1 (red, Figure C) foci at 30 min after irradiation with 6 Gy with confocal microscopy revealing discrete and more diffuse foci formation in HCC1395 compared to wildtype cells (MCF10A). Scale bar = 5 μm. **D**. Enlarged area of single nuclei from **B**, **C** for quantitative evaluation. Note the smaller area and fuzzy appearance of foci in the HCC1395 cell line. **E**. Analysis of foci area in HCC1395 and wildtype cells (MCF10A) for γH2AX, 53P1 and MDC1. For each, areas of 60–90 foci were analysed. Data are represented as mean & SEM. *p < 0.05.

**Figure 4:**
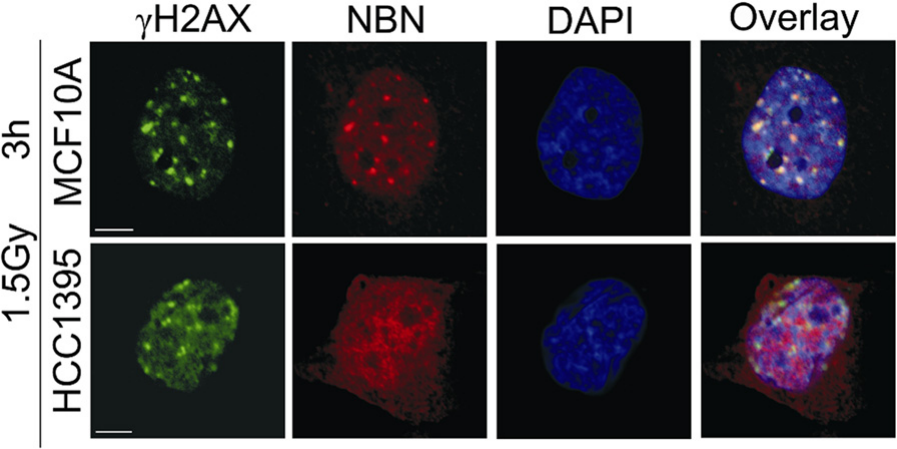
**NBN foci formation.** Double immunostaining of γH2AX (green) and NBN (red) foci in HCC1395 and wildtype cells (MCF10A). Cells were fixed 3 h after irradiation with 1.5 Gy. DNA was counterstained with DAPI. Note the reduced appearance and brightness of yH2AX foci and the absence of NBN foci in the HCC1395 cell line. Scale bar = 5 μm.

Because the cellular radiation response is mediated through a cooperation of NBN with ATM, we investigated the radiation-induced kinase activity of ATM in HCC1395 cells. There was little difference between the NBN mutant cells and MCF10A in the radiation-induced phosphorylation of SMC1, CHEK2 or KAP1, respectively (Figure 2C). This was independent of total protein levels as exemplified for KAP1 in Additional file 5: Figure S4. While HCC1395 cells showed a somewhat higher basic level of phosphorylation in the unirradiated state, the fact that irradiated cells reached the same level of phosphorylation as wildtype cells indicated a largely unaffected ATM kinase activity towards these major substrates within the dose range studied (0.5 – 6 Gy; Figure 2C).In order to investigate the molecular basis of radiation sensitivity in HCC1395 cells in more detail, we analysed radiation induced foci by immunocytochemical analyses of three repair proteins γH2AX, 53BP1 and MDC1. In HCC1395 cells, foci for these three proteins were generally less intense and appeared smaller than in MCF10A cells. In a quantitative approach of counting foci with conventional fluorescence microscopy on MCF10A, HCC1395 and HCC1937 under the same conditions, the percentages of γH2AX and MDC1 foci were significantly reduced in HCC1395 cells compared to wildtype MCF10A at 30 min after irradiation with either 1.5 or 6 Gy, whereas they were at least as high as wildtype in HCC1937 cells in which they also persisted at 24 hours after irradiation (Figure 3A). A closer inspection using confocal laser microscopy revealed a smaller and a more fuzzy appearance of foci in HCC1395 cells compared with MCF10A. A significant reduction in the size of γH2AX foci was noted as determined by measuring the γH2AX foci area in HCC1395 (Figures 3B-E). When staining against NBN itself, wildtype MCF10A cells showed multiple discrete NBN foci after irradiation whereas no such NBN foci were detected in HCC1395 cells, indicating a strongly reduced capability of p.R215W NBN to accumulate in radiation-induced foci (Figure 4).

Because NBN may facilitate homologous recombinational repair [25,26], and PARP1 activity has been reported to be aberrantly high in NBS cells [27], we speculated that PARP1 inhibition may be effective in HCC1395 cells. We therefore treated HCC1395 cells with different doses of olaparib, a known PARP1 inhibitor used in clinical trials, and determined its effect on cellular viability and proliferation using real time monitoring of cellular impedance. Olaparib significantly decreased the cell index at doses as low as 0.2 μM. At this concentration, no such effect of olaparib was observed in MCF10A (Additional file 6: Figure S5), nor in HCC1937, suggesting a particular sensitivity of HCC1395 cells with their combined mutations in BRCA1 and NBN towards PARP1 inhibition.

## Discussion

In a search for breast cancer cell lines with deficiencies in the MRN-ATM pathway, and thus defective DNA double strand break response, we here describe the triple-negative breast cancer cell line, HCC1395, as being mutated in the *NBN* gene. HCC1395 is a known breast cancer cell line and has been included in previous genome-wide sequencing studies [22-24] but the *NBN* mutation p.R215W had not been uncovered. At the time of submission, database mining of available cancer cell lines in the COSMIC database (http://cancer.sanger.ac.uk/cosmic/) failed to identify any other line with the p.R215W mutation, and also did not reveal any other breast cancer cell line with a coding *NBN* mutation, so that HCC1395 may provide a unique source for studying the molecular consequences of NBN deficiency. The observation that p.R215W had already been present as a germ-line mutation in the heterozygous patient and underwent loss of heterozygosity in the tumour would be in line with the view that it represents a driver rather than a passenger mutation during tumorigenesis, and is consistent with the previously reported loss of heterozygosity in breast carcinomas harboring the NBN*657del5 mutation [14].

One consequence of the p.R215W mutation appears to be a somewhat reduced stability of the NBN protein which was expressed at a lower level than would be expected for a hemizygous situation. This did not affect the levels of MRE11A or RAD50, consistent with the report that BRCT mutations in NBN do not interfere with MRN complex formation in vitro [28]. The reduced level of p.R215W mutant protein is also consistent with previous results from lymphoblastoid cells of p.R215W/ c.657del5 compound heterozygous NBS patients which similarly showed markedly reduced nibrin levels [19,20]. Nevertheless, the residual NBN protein was easily detectable and, at the reduced level, appeared to undergo a rapid mobility shift after irradiation as expected for normally activated NBN. An unimpaired phosphorylation shift of NBN after irradiation has similarly been found in the compound heterozygous NBS patient cells [20] and may indicate that the p.R215W substitution does not strongly disturb the ATM-mediated phosphorylation of NBN at the more distant serine-278 and serine-343 sites. Furthermore, although NBN has been shown to be required for full ATM activation after irradiation [11,29] and particularly for its phosphorylation of SMC1 [30,31] the p.R215W mutant did not seem to strongly disturb the ATM-mediated phosphorylation of three different substrates including SMC1 after irradiation in HCC1395 cells, suggesting that it still functions in the intra-S-phase checkpoint. This appears to be inconsistent with a recent study that reports impairment of ATM-mediated phosphorylation through p.R215W in retrovirally transduced fibroblasts [32] but is in agreement with the normal behaviour of other BRCT mutants [28] and is supported by the flow cytometric analyses of HCC1395 cells which did not uncover marked cell cycle abnormalities, such as a rapid G2 accumulation due to radioresistant DNA synthesis, after up to 72 hours. In line with this, previous work had indicated that the BRCT domain of NBN is not required for inhibition of DNA synthesis [11] or for cell cycle checkpoint regulation which rather may be mediated by the FHA domain that is preserved in the p.R215W mutant [33]. The retained activation of the cohesion protein, SMC1, may help to suppress chromatid-type aberrations [34], and such aberrations are indeed lacking in p.R215W/c.657del5 compound heterozygous NBS patients [20].

The BRCT domain, initially described in BRCA1, is a phosphoprotein binding module that is common in several DNA damage responsive proteins [35,36] and, in case of NBN, has been implicated in the binding of phosphorylated H2AX [9]. Phosphorylation of H2AX at Ser139 is among the first events of the repair of double strand breaks [37]. The gross accumulation of γH2AX into foci, like those of MDC1 and 53BP1, appeared to be incomplete in HCC1395 cells although smaller foci could well be detected. It is possible that, after an initial phosphorylation of these repair proteins, p.R215W mutant NBN fails to properly localize to γH2AX and to recruit or stabilize ATM which may be required for a second wave of phosphorylation and focus extension. Our results support a previous study, using a targeted mutagenesis approach [21], which showed that mutation R215W prevented the binding of NBN to γH2AX following radiation, and suggests a functional relevance for this mutation in protein-protein interactions. This view is further supported by molecular modelling of the tandem BRCT domain of NBN in which the R215 residue, similar to the R1989 residue in MDC1, appears to direct the relative orientation of both BRCT domains and thereby regulate γH2AX recognition [21]. In a study of additional single mutations of either the FHA or BRCT domains, only a small fraction of mutant NBN accumulated along DNA damage tracks labelled by γH2AX after irradiation [33], and given that only one BRCT domain had been mutated, it seems plausible that p.R215W may have a stronger effect than the p.K160R mutant used in that study.

The results obtained in HCC1395 might have more general implications for breast cancer as they strongly indicate functional and clinical relevance for a missense mutation that is not uncommon among Europeans. p.R215W has not been studied in the homozygous state, thus far, because no homozygous individual has been identified despite the modestly high prevalence of around 1/200 in some populations [19,20]. As suggested by Seemanova and co-workers, homozygosity for the p.R215W mutation may lead to early embryonic lethality, albeit the data here clearly indicate that some residual expression and function is associated with this mutation. Of note, it appears from Figure 2C as well as based on Figure 2B that the hemizygous HCC1395 line may be suffering considerable damage even without additional endogenous exposure (higher levels of pSMC1, and Chk2 phosphorylation and PARP cleavage in 0 Gy irradiated samples) and this finding may be related to its poor growth overall. These features are also commonly observed for classical NBN mutant lines. Heterozygosity for p.R215W, as observed for the patient BL1395, has been previously associated with breast cancer in some, though not all, study populations [15,19,38], and it is possible that p.R215W plays a predisposing role also for other malignancies [39]. At present, the functional impact of the p.R215W NBN defect in breast cancer susceptibility is still controversial, but evidence is accumulating that this mutation might predispose individuals to disease.

It is also possible that p.R215W exerts such a predisposing role as a modifier of penetrance for additional mutations such as in *BRCA1*. In fact, the HCC1395 cell line was also found to harbor a truncating *BRCA1* mutation. This mutation, however, is unlikely to explain the reduced NBN levels or impaired formation of repair foci considering that BRCA1 is not upstream of NBN and was also mutated in the HCC1937 cell line which was normal in *NBN* and proficient in the formation of repair foci. Although we cannot formally exclude the possibility that a *BRCA1* mutation augments the effect of *NBN* p.R215W on NBN levels and radiation-induced foci formation, the functional BRCA1 deficiency does not cause these effects as is also evidenced by the observations of normal NBN levels and foci formation in other cell lines such as HCC38 or HCC1806 which are functionally deficient in BRCA1 [40,41]. However, the presence of a BRCA1 or BRCA2 mutation has been shown to affect sensitivity towards ionising radiation [42] as well as towards PARP1 inhibition [43,44], so that these endpoints were not specific for the NBN*R215W mutant. Increased sensitivity to PARP1 inhibition has previously been reported for NBN deficient cells, and mutations in other proteins that affect HR, besides BRCA1/2, may also show some PARP1 inhibitor sensitivity [45,46]. On the other hand, increased sensitivity to PARP1 inhibition is a known feature of *BRCA1* and *BRCA2* mutant cells, and since we found HCC1395 cells to harbour a *BRCA1* truncation in the hemizygous and a *BRCA2* mutation in the heterozygous state, it is likely that the high olaparib sensitivity is a combinatorial result and we could not fully determine in the present study to what extent these observations were due to functional deficiency in NBN, BRCA1, BRCA2 or perhaps other deficiencies. These results emphasize the need to fully explore the mutational genotypes of cell lines as well as of primary tumours before final conclusions can be drawn. The slow proliferation of HCC1395 cells and their higher genomic instability and higher apoptotic activity under basal conditions as indicated in Figures 2B, C and Additional file 6: Figure S5 may further impact on these endpoints and, given the heterogeneous background of breast tumours, additional studies of p.R215W carriers will be needed to fully elucidate this aspect and clarify whether they could perhaps benefit from treatment with PARP1 inhibitors.

## Conclusion

We report for the first time the identification and characterization of a breast cancer cell line containing a NBN mutation that affects its function, providing a potentially useful cellular model for studying defective NBN protein within a mutant BRCA1 background. The data indicate that NBN*p.R215W is an unstable protein that fails to be recruited into foci after irradiation and also impairs the propagation of γH2AX repair foci. Our results furnish evidence for the functional impact of the p.R215W substitution, a suspected cancer susceptibility allele in Europeans.

## Abbreviations

NBS: Nijmegen breakage syndrome; MRN: MRE11-RAD50-NBN; BRCT: Breast-cancer carboxy-terminal domain; DMSO: Dimethylsulfoxide; PFA: Paraformaldehyde; PBS: Phosphate-buffered saline; EGTA: Ethylene glycol tetraacetic acid; EDTA: Ethylenediamine tetraacetic acid; PMSF: Phenylmethanesulfonylfluoride.

## Competing interests

All authors declare to have no financial or non-financial competing interests.

## Authors’ contributions

BSH, NB and TD participated in the conception and design of the study. Data acquisition and analyses were performed by BSH, NB, XX, MN and BW. Data acquisition was supported by PH, TWPS and HC. The manuscript was drafted by BSH, NB, MN, XX, BW and TD. All authors read and approved the final manuscript.

## Pre-publication history

The pre-publication history for this paper can be accessed here:

http://www.biomedcentral.com/1471-2407/14/434/prepub

## Supplementary Material

Additional file 1: Table S1 Primer pairs used for amplification and sequencing the coding region of *NBN* and selected regions of *BRCA1* (exon 20), *BRCA2* (exon 11) and *TP53* (exon 5). Click here for file

Additional file 2: Figure S1 Validation by direct sequencing of additional mutations in *BRCA1*, *BRCA2* and *TP53* in HCC1395 breast cancer cells. Direct sequencing of selected regions of *BRCA1*, *BRCA2* and *TP53* in HCC1395 BL lymphoblastoid cells (top) and HCC1395 breast cancer cells (bottom) to validate reported mutations and confirm the identity of HCC1395 cells. Mutated positions in *BRCA1* (left panel), *BRCA2* (middle panel) and *TP53* (right panel) are indicated by an arrow and the mutated codon is boxed. Like the *NBN* mutation p.R215W, the *BRCA1* mutation p.Y1751X appears heterozygous in HCC1395 BL lymphoblasts but homoallelic in HCC1395 breast cancer cells, whereas *TP53* and *BRCA2* mutations were somatic events (with *BRCA2* mutated only in the heterozygous state) in HCC1395 breast cancer cells. Click here for file

Additional file 3: Figure S2 Assessment of cellular radiosensitivity in the colony formation assay (higher dose experiments). Cellular radiosensitivity of p.R215W mutant cells (HCC1395) compared with wild type breast epithelial cells (MCF10A) as measured by the colony formation assay after irradiation at doses of 2, 4, or 6 Gy. The surviving fraction is presented as the mean value with SEM from at least 3 independent experiments. Click here for file

Additional file 4: Figure S3 Cell cycle analysis by flow cytometry in HCC1395 breast cancer cells. Flow cytometric analyses of S-, G1 and G2 cell population in wildtype cells (MCF10A) (A), HCC1395 (B) and HCC1937 (C) after irradiation with 6 Gy and different time points (24 hrs, 48 hrs, 72 hrs, 96 hrs). Data represented as Mean & SEM. Data for 24 hrs, 48 hrs and 72 hrs: n = 3; data for 96 hrs: n = 1. Click here for file

Additional file 5: Figure S4 Immunoblot analysis of total and phosphorylated KAP1 in MCF10A and HCC1395 cells treated with different doses of irradiation. Immunoblot analysis of radiation-induced ATM signalling in HCC1395 cells compared with MCF10A. Cells were untreated or irradiated with 0.5, 1, 2, 4 or 6 Gy as indicated. Protein extracts were prepared 30 min after irradiation and were analysed through Western blotting for their immunoreactivity towards the phosphorylated form of KAP1 (p824, upper panel) and total KAP1 (bottom panel), respectively. β-actin served as the loading control. Click here for file

Additional file 6: Figure S5 Response to PARP1 inhibition in X-Celligence impedance measurements. X-Celligence impedance measurements of the p.R215W mutant cell line (HCC1395) were performed over three days after addition of the PARP1 inhibitor olaparib at different concentrations (0.2 μM, 1 μM, 5 μM; increasing concentrations from top to bottom) and compared with the DMSO only control. Top: HCC1395 NBN p.R215W mutant cell line; bottom: MCF10A cell line for comparison. Data are presented as mean values & SEM from quadruplicates. Click here for file
