# Supplementary material for: Functional deficiency of NBN, the Nijmegen breakage syndrome protein, in a p.R215W mutant breast cancer cell line
Source: BMC Cancer. 2014 Jun 13;14:434. doi: 10.1186/1471-2407-14-434 (PMC4085727; doi:10.1186/1471-2407-14-434)
Supplement: Additional file 1: Table S1 — Primer pairs used for amplification and sequencing the coding region of NBN and selected regions of BRCA1 (exon 20), BRCA2 (exon 11) and TP53 (exon 5). [file 1471-2407-14-434-S1.doc]

**Supplementary Table 1:**

Primers used for *NBN* sequencing:

NBN1i-5 5´-TCA TCC AAG GCA GCC TGC GT-3´

NBN1i-3 5´-TGC CAT ACA GCG TAC TCG CC-3´

NBN2i-5 5´-CTT TGA TAG CCT TCA GTG AG-3´

NBN2i-3 5´-CTC TCT CTC ACA TAC AAA CC-3´

NBN3i-5 5´-CAG TAA TTG TTG TCT GCC GT-3´

NBN3i-3 5´-AGG ATT TGG CTG AAA CAA AG-3´

NBN4i-5 5´-GCT TAA TGA TGA GGA ACT GA-3´

NBN4i-3 5´-CCT AAA TGG TAT ACA AAG GG-3´

NBN5i-5 5´-TTA TGG ATG TAA ACA GCC TC-3´

NBN5i-3 5´-TAC CGA ACT ATA ACA CAG CA-3´

NBN6i-5 5´-CCT TTT GAG TGT CAG ATA GTC-3´

NBN6i-3 5´-TGA AAT ACG TTA ACA ACT ACT G-3´

NBN7i-5 5´-TCA AGA AGT AGC ACC AAG TC-3´

NBN7i-3 5´-AAT TGC TTG AAC CCA GAA GG-3´

NBN8i-5 5´-GAG GTT GCT TTA TCT TGA CA-3´

NBN8i-3 5´-CCC TAG CAA GTA TAT AGA TA-3´

NBN9i-5 5´-CTT AGC ATG GTA TAG TCT AA-3´

NBN9i-3 5´-CTC AAG AGA CAA CCT GAT AA-3´

NBN10i-5 5´-TGC TTT CTT GGG ATG GTA AA-3´

NBN10i-3 5´-GCA GAA GCA TAC TTA ATC AG-3´

NBN11i-5 5´-ATG GTT ACT TAG CTG TGT TC-3´

NBN11i-3 5´-TAA TGG ATG CTC ATA CTG TC-3´

NBN12i-5 5´-ATG CCT GGT CAT ACA TAA CA-3´

NBN12i-3 5´-AAT TGA TGA GAT GAC AGT CC-3´

NBN13i-5 5´-AGA TTC CCA AAT GAC AAG TG-3´

NBN13i-3 5´-AGT TCA TAT CCT TCC TAG AG-3´

NBN14i-5 5´-AAC ATC TTT GGC ACT TAT GC-3´

NBN14i-3 5´-AGA AGA ATT TGC TTG AAG GC-3´

NBN15i-5 5´-CTA TTG GTT GTC TTT GAG TG-3´

NBN15i-3 5´-ATT TCA CAC AAT TCG GGA AC-3´

NBN16ia-5 5´-TCA TTC CCA TCC TAT TTG CC-3´

NBN16ia-3 5´-TGG AAG GGT GAC TTT AGT CT-3´

NBN16ib-5 5´-AGG TAA AGA CTA AAG TCA CC-3´

NBN16ib-3 5´-TGT TTG ATG AAG TCT CCA CA-3´

NBN16ic-5 5´-AGT ACT AGA AAC TGA AGA CC-3´

NBN16ic-3 5´-ATT TGG AAG GTG AGA GTG AT-3´

NBN16id-5 5´-GTA AAC AGA AGC AAC AGA AG-3´

NBN16id-3 5´-GGC AAG GTA ATT TAA TGA GG-3´

Primers used for *BRCA1* sequencing:

BR20i5 5’- TCT CTT ATC CTG ATG GGT TG - 3’

BR20i3 5’- GGG AAT CCA AAT TAC ACA GC -3’

Primers used for *BRCA2* sequencing:

BS15C 5’ - CAG AGA GGC CTG TAA AGA CCT TG - 3’

BS16 5’ - AAA TAA TTT CCT ACA TAA TCT GCA G- 3’

Primers used for *TP53* sequencing:

TP53_5F 5´-CgT CTT CCA gTT gCT TTA TCT g-3´

TP53_6R 5´-TCA CCT ggA ggg CCA CTg AC-3´
