# Supplementary figures and images for: Functional deficiency of NBN, the Nijmegen breakage syndrome protein, in a p.R215W mutant breast cancer cell line
Source: BMC Cancer. 2014 Jun 13;14:434. doi: 10.1186/1471-2407-14-434 (PMC4085727; doi:10.1186/1471-2407-14-434)

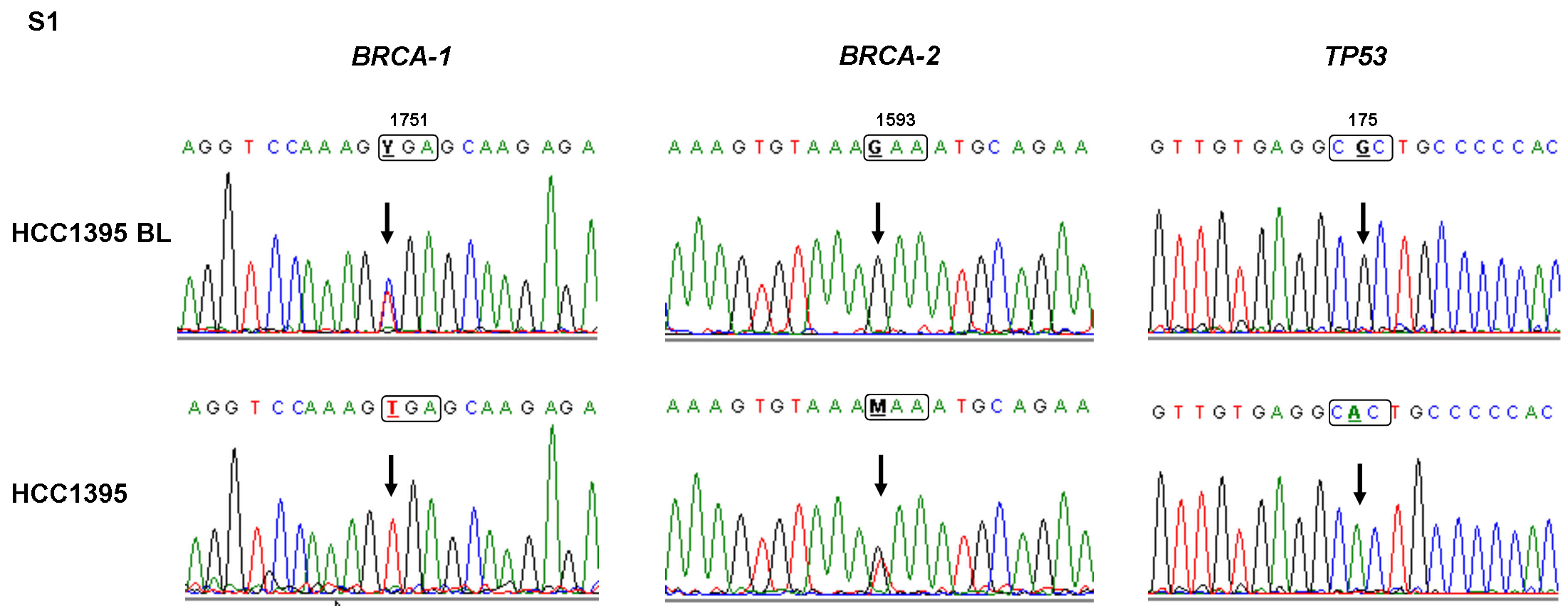

Supplement: Additional file 2: Figure S1 — Validation by direct sequencing of additional mutations in BRCA1, BRCA2 and TP53 in HCC1395 breast cancer cells. Direct sequencing of selected regions of BRCA1, BRCA2 and TP53 in HCC1395 BL lymphoblastoid cells (top) and HCC1395 breast cancer cells (bottom) to validate reported mutations and confirm the identity of HCC1395 cells. Mutated positions in BRCA1 (left panel), BRCA2 (middle panel) and TP53 (right panel) are indicated by an arrow and the mutated codon is boxed. Like the NBN mutation p.R215W, the BRCA1 mutation p.Y1751X appears heterozygous in HCC1395 BL lymphoblasts but homoallelic in HCC1395 breast cancer cells, whereas TP53 and BRCA2 mutations were somatic events (with BRCA2 mutated only in the heterozygous state) in HCC1395 breast cancer cells. [file 1471-2407-14-434-S2.tiff]

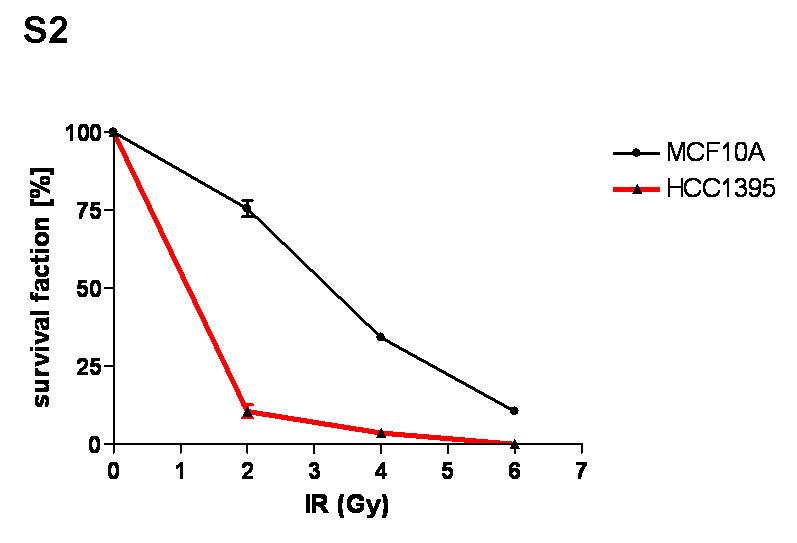

Supplement: Additional file 3: Figure S2 — Assessment of cellular radiosensitivity in the colony formation assay (higher dose experiments). Cellular radiosensitivity of p.R215W mutant cells (HCC1395) compared with wild type breast epithelial cells (MCF10A) as measured by the colony formation assay after irradiation at doses of 2, 4, or 6 Gy. The surviving fraction is presented as the mean value with SEM from at least 3 independent experiments. [file 1471-2407-14-434-S3.tiff]

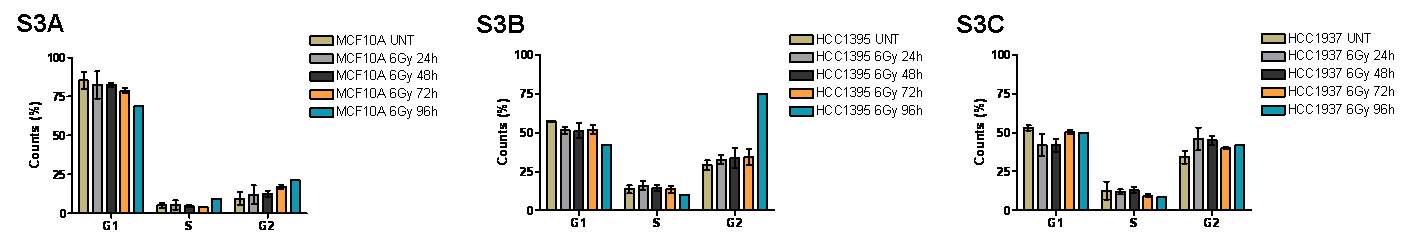

Supplement: Additional file 4: Figure S3 — Cell cycle analysis by flow cytometry in HCC1395 breast cancer cells. Flow cytometric analyses of S-, G1 and G2 cell population in wildtype cells (MCF10A) (A), HCC1395 (B) and HCC1937 (C) after irradiation with 6 Gy and different time points (24 hrs, 48 hrs, 72 hrs, 96 hrs). Data represented as Mean & SEM. Data for 24 hrs, 48 hrs and 72 hrs: n = 3; data for 96 hrs: n = 1. [file 1471-2407-14-434-S4.tiff]

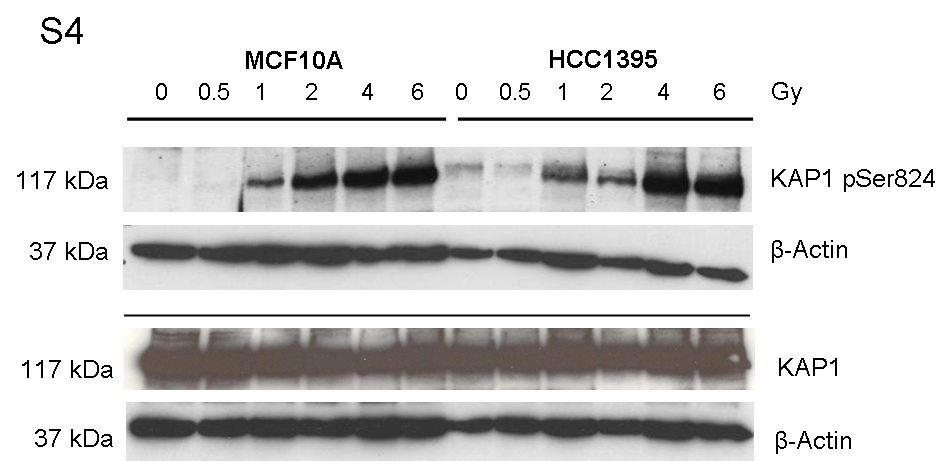

Supplement: Additional file 5: Figure S4 — Immunoblot analysis of total and phosphorylated KAP1 in MCF10A and HCC1395 cells treated with different doses of irradiation. Immunoblot analysis of radiation-induced ATM signalling in HCC1395 cells compared with MCF10A. Cells were untreated or irradiated with 0.5, 1, 2, 4 or 6 Gy as indicated. Protein extracts were prepared 30 min after irradiation and were analysed through Western blotting for their immunoreactivity towards the phosphorylated form of KAP1 (p824, upper panel) and total KAP1 (bottom panel), respectively. β-actin served as the loading control. [file 1471-2407-14-434-S5.tiff]

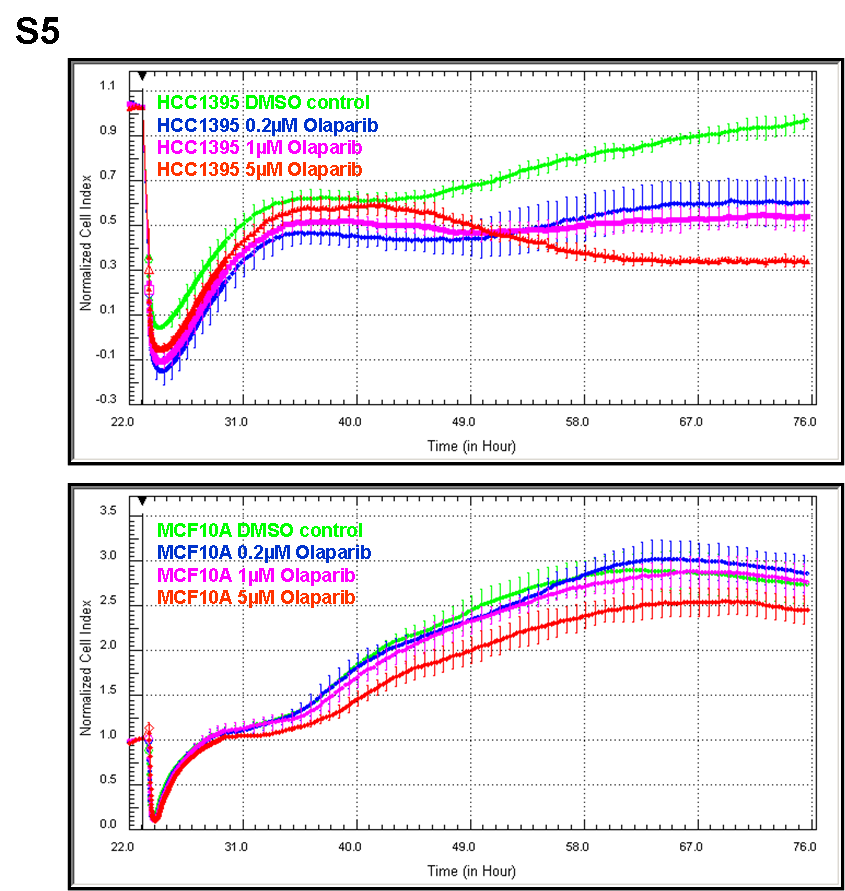

Supplement: Additional file 6: Figure S5 — Response to PARP1 inhibition in X-Celligence impedance measurements. X-Celligence impedance measurements of the p.R215W mutant cell line (HCC1395) were performed over three days after addition of the PARP1 inhibitor olaparib at different concentrations (0.2 μM, 1 μM, 5 μM; increasing concentrations from top to bottom) and compared with the DMSO only control. Top: HCC1395 NBN p.R215W mutant cell line; bottom: MCF10A cell line for comparison. Data are presented as mean values & SEM from quadruplicates. [file 1471-2407-14-434-S6.tiff]
